# Target-Site and Non-target-Site Resistance Mechanisms Confer Multiple and Cross- Resistance to ALS and ACCase Inhibiting Herbicides in *Lolium rigidum* From Spain

**DOI:** 10.3389/fpls.2021.625138

**Published:** 2021-02-04

**Authors:** Joel Torra, José María Montull, Andreu Taberner, Nawaporn Onkokesung, Neil Boonham, Robert Edwards

**Affiliations:** ^1^Department d’Hortofructicultura, Botànica i Jardineria, Agrotecnio, Universitat de Lleida, Lleida, Spain; ^2^Agriculture, School of Natural and Environmental Sciences, Newcastle University, Newcastle upon Tyne, United Kingdom

**Keywords:** glutathione-s-transferase, ACCase inhibitor, ALS inhibitor, cytochrome P450 monooxygenase, enhanced metabolism, photosystem II inhibitor, thiocarbamate herbicide, very-long-chain fatty acids biosynthesis inhibitor

## Abstract

*Lolium rigidum* is one the worst herbicide resistant (HR) weeds worldwide due to its proneness to evolve multiple and cross resistance to several sites of action (SoA). In winter cereals crops in Spain, resistance to acetolactate synthase (ALS)- and acetyl-CoA carboxylase (ACCase)-inhibiting herbicides has become widespread, with farmers having to rely on pre-emergence herbicides over the last two decades to maintain weed control. Recently, lack of control with very long-chain fatty acid synthesis (VLCFAS)-inhibiting herbicides has been reported in HR populations that are difficult to manage by chemical means. In this study, three Spanish populations of *L. rigidum* from winter cereals were confirmed as being resistant to ALS- and ACCase-inhibiting herbicides, with broad-ranging resistance toward the different chemistries tested. In addition, reduced sensitivity to photosystem II-, VLCFAS-, and phytoene desaturase-inhibiting herbicides were confirmed across the three populations. Resistance to ACCase-inhibiting herbicides was associated with point mutations in positions Trp-2027 and Asp-2078 of the enzyme conferring target site resistance (TSR), while none were detected in the ALS enzyme. Additionally, HR populations contained enhanced amounts of an ortholog of the glutathione transferase phi (F) class 1 (GSTF1) protein, a functional biomarker of non-target-site resistance (NTSR), as confirmed by enzyme-linked immunosorbent assays. Further evidence of NTSR was obtained in dose-response experiments with prosulfocarb applied post-emergence, following pre-treatment with the cytochrome P450 monooxygenase inhibitor malathion, which partially reversed resistance. This study confirms the evolution of multiple and cross resistance to ALS- and ACCase inhibiting herbicides in *L. rigidum* from Spain by mechanisms consistent with the presence of both TSR and NTSR. Moreover, the results suggest that NTSR, probably by means of enhanced metabolism involving more than one detoxifying enzyme family, confers cross resistance to other SoA. The study further demonstrates the urgent need to monitor and prevent the further evolution of herbicide resistance in *L. rigidum* in Mediterranean areas.

## Introduction

*Lolium rigidum* (Gaud.) is a genetically diverse, cross-pollinating and globally distributed weed species, that has evolved resistance to herbicides acting on many sites of action (SoA) around the world (Yu and Powles, 2014). To date, *L. rigidum* has evolved resistance to 14 different herbicide SoA around the globe (Heap, 2020). In Europe, resistance to acetyl CoA carboxylase (ACCase), acetolactate synthase (ALS), 5-enolpyruvylshikimate-3-phosphate synthase (EPSPS), glutamine synthase, and protoporphyrinogen oxidase (PPO) inhibiting herbicides has been reported in *L. rigidum* associated with winter cereals, vineyards and orchards in the southern part of the continent (De Prado et al., 2005; Cirujeda and Taberner, 2010; Kaloumenos et al., 2012; Atanackovic et al., 2015; Fernandez-Moreno et al., 2017; Fernández-Moreno et al., 2017; Heap, 2020). As such, multiple, or cross-resistance to several SoA is now widespread in southern Europe.

In rainfed cereals from Spain, the first case of a herbicide resistant (HR) *L. rigidum* Gaud. population was reported in the north-eastern region, with resistance reported to chlortoluron (PSII inhibiting herbicide) and diclofop-methyl (ACCase inhibiting herbicide) in 1995 (Taberner et al., 1995). Failures in the control of *L. rigidum* with chlorsulfuron (ALS inhibiting herbicide) were subsequently reported in the river Duero region in Castilla-León (de la Carrera et al., 1999). ACCase, ALS, and PSII inhibitors resistance is now widespread throughout the Spanish winter cereal cropping system (Loureiro et al., 2017), with 75% of *L. rigidum* populations in Catalonia exhibiting HR to these SoA (Loureiro et al., 2010, 2017). The resistance problem is becoming even more complex, with HR reported toward other graminicides used in canola, including clethodim and fluazifop-butyl (Cirujeda and Taberner, 2003).

In response to HR toward ACCase, PSII, and ALS inhibiting, since 2000 there has been an increased reliance on pre-emergence soil-applied herbicides such as prosulfocarb from the thiocarbamate family (Group 15 HRAC/WSSA, inhibition of fatty acid elongase) and less often trifluralin from the dinitroaniline family (Group 3, inhibition of microtubule assembly). Unfortunately, trifluralin has been banned in the European Union since 2009, primarily due to toxicity in aquatic environments (Commission Decision 2010/455/EU; 26 June 2010). This has placed greater pressure on herbicides from groups 15, such as oxyacetamides (flufenacet) or chloroacetamides (metazachlor) and group 3 (propyzamide). These are the only available MoA, excluding non-selective herbicides, still available to control problematic *L. rigidum* in Spanish winter cereals and rotational crops.

Resistance to ALS and ACCase inhibiting herbicides can be caused by a mutation in the genes encoding the respective proteins, which is termed target-site resistance (TSR). The mutations at specific positions on ALS or ACCase enzyme commonly confer resistance to specific herbicide chemistries. In *L. rigidum*, amino acid substitutions at positions Pro197 and Trp574 (among three other positions) in the ALS enzyme are the common mutations associated with the resistance to sulfonylurea (SU) ALS-inhibiting herbicides, while the second position to imidazolinone (IMI) too (Yu et al., 2008; Tranel et al., 2020). The amino acid substitutions at positions Ile1781, Trp2027, Ile2041, and Asp-2078 (among two other positions) of ACCase, have been frequently identified to confer resistance to aryloxyphenoxypropionates (APP) and cyclohexanediones (CHD) herbicides (Kaundun, 2014; Murphy and Tranel, 2019). Besides TSR, this species can also develop non-target-site resistance (NTSR) to multiple herbicide chemistries through various mechanisms including increased herbicide detoxification, also referred to as enhanced metabolic resistance (EMR). EMR has been recorded in *L. rigidum* both for ACCase and ALS inhibiting herbicides (Gaines et al., 2020). Enhanced herbicide metabolism is associated with the increased expression of detoxifying enzymes, including cytochrome P450 monooxygenases (CYP450) and glutathione *S-*transferases (GSTs) (Ghanizadeh and Harrington, 2017). In EMR *L. rigidum* populations, the CYP450 family has been identified as one of the important enzyme family conferring resistance to ACCase and ALS inhibiting herbicides (Gaines et al., 2020).

GSTs are known to detoxify herbicides through catalyzing their conjugation with glutathione (GSH) (Cummins et al., 2013; Gaines et al., 2020). In *Alopecurus myosuroides*, the constitutive level of a glutathione transferase phi (F) class 1 protein (*AmGSTF1*) was significantly higher in multiple herbicide resistance populations than in sensitive populations (Comont et al., 2020). The biochemistry of *AmGSTF1* and its transgenic expression in *Arabidopsis thaliana* showed that this protein has a functional role in regulating NTSR that extends beyond the glutathione conjugation of herbicides (Cummins et al., 1999, 2013). Furthermore, the increased expression of the orthologous transcript and protein *LrGSTF1* in *L. rigidum* populations is also associated with NTSR (Cummins et al., 2013; Busi et al., 2018). Together, this information indicates that the level of the *AmGSTF1* ortholog, *LrGSTF1*, could be used as a biomarker to identify the existence of NTSR in *L. rigidum* populations.

In HR *L. rigidum* populations from rainfed cereals in Spain, the resistance mechanisms to ACCase inhibiting herbicides were previously studied (De Prado et al., 2005), while those conferring resistance to ALS have not been reported. Moreover, cross- and multiple herbicide resistance across different chemistries by EMR and the enzymes involved have not been reported. Furthermore, the first cases of failures in *L. rigidum* control with other herbicide SoA apart from ALS or ACCase inhibitors were reported in Catalonia in 2010 (Comité para la Prevención de Resistencias a Herbicidas [CPRH], 2015). Therefore, the identification of the associated herbicide resistance mechanisms is now required for tailoring the weed management of multiple HR *L. rigidum* populations in winter cereals in Spain.

In this study, the potential for TSR and NTSR mechanisms to evolve toward ALS- and ACCase inhibiting herbicides was investigated in three populations from Spain. Also, resistance to thiocarbamate herbicides was characterized in whole plant studies. Additionally, we report on the response of these populations to PSII-inhibiting herbicides, fatty acid elongase inhibiting herbicides, and fatty acid synthase plus carotenoid biosynthesis inhibiting herbicides.

## Materials and Methods

### Plant Material

*Lolium rigidum* populations used in this study came from winter cereal fields where lack of control after prosulfocarb treatments was reported. All the fields were located in North-Eastern Spain, with prosulfocarb being the only herbicide applied over several years (≥5 years). This herbicide is usually applied when ACCase, ALS, and PSII inhibiting herbicides do not provide sufficient efficacy. Populations, both resistant (R) and susceptible (S), were collected during 2014 and 2015 (Table 1). The population considered as S had never been exposed to herbicides.

**TABLE 1:** *Lolium rigidum* populations from Spanish winter cereal fields, location, year of seed collection, and potential resistance profile.

**Biotype**	**Locality**	**Year of seed collection**	**Profile**
ES-MHR 1	Calonge de Segarra	2015	Multiple Herbicide Resistant
ES-MHR 2	Calonge de Segarra	2015	Multiple Herbicide Resistant
ES-MHR 3	Calaf	2014	Multiple Herbicide Resistant
ES-S 1	Ballobar	2014	Susceptible

Seeds were incubated at 40°C for 3 weeks to break seed dormancy and then stratified at 4°C for 7 days to promote and synchronize germination. After 7 days, germination plates were moved to the growth cabinet (16/8 h light, 18°C) for 1 week before transplanting four germinated seedlings per pot.

### Herbicide Screening

In this experiment, 10 seeds per pot were sown in 7 × 7 × 9 cm pots filled with a mixture of soil, perlite and peat (4/1/2 v/v/v). Pots were placed in a greenhouse located in the University of Lleida, north-eastern Spain (41°37′N, 0°38′E) and kept with a light regime of 14 h at 25°C, provided by supplementary lighting, and 10 night hours at a minimum temperature of 10°C. Irrigation was at demand, daily, through a shower system without water reuse, with fertilization as required. One week after sowing PRE herbicides were applied at the BBCH stage of 00-09 (Zadoks et al., 1974). To test herbicides in POST, after seedling emergence the plants per pot were thinned to four, and then were sprayed at the BBCH stages of 11-12 or 13-21 (Zadoks et al., 1974; Table 2). Herbicide screening was conducted as described in Table 2.

**TABLE 2:** Trade name, company, active ingredients, and sites of action of herbicides tested on the *Lolium rigidum* populations.

**Trade Name**	**Company**	**Active ingredients**	**Site of Actions**	**BBCH**	**Rates (g ha^–1^)**
Atlantis	Bayer CropScience	Mesosulfuron ++ iodosulfuron	ALS inhibitor	13–21	4+1, 8+2, 15+3
Broadway Star	Dow AgroSciences	Pyroxsulam + florasulam	ALS inhibitor	13-21	9+2, 19+4, 38+8
Iloxan	Bayer CropScience	Diclofop-methyl	ACCase inhibitor	13-21	720, 360, 180
Select	FMC	CLETHODIM	ACCase inhibitor	13-21	24, 48, 96
Clortolurex	Adama Agriculture	Chlortoluron	PS II inhibitor	11-12	375, 750, 1500
Herold	Bayer CropScience	Flufenacet + diflufenican	Groups 15+12	00-09	60+30, 120+60, 240+120
Auros	Syngenta Agro	Prosulfocarb	Group 15	00-09	1,000, 2,000, 4,000

*All companies were located in Spain. 15, inhibitors of the fatty acid synthase; 12, carotenoid biosynthesis inhibitors; group 15, fatty acid elongase inhibitor. BBCH: growth stage at which herbicides were applied. Three doses were applied per herbicide; being the highest maximum registered dose in Spain.*

The active ingredient florasulam in Broadway predominately provides broad-leaf weed control. Therefore, for the purposes of this screening, the efficacy of the other active ingredient in Broadway, pyroxsulam, was evaluated against *L. rigidum*. In Herold, the active ingredient flufenacet has the main activity against *L. rigidum*, as compared with diflufenican, and was the subject of this study. Due to the large number of herbicides/populations to be tested and seed availability, only three rates per herbicide were used (Table 2). Herbicides were applied using a precision bench sprayer delivering 200 L ha^–1^, at a pressure of 200 kPa and equipped with two Hardi Flat fan nozzles. 28 days after treatment plants were harvested (above ground) and fresh weight measured. Experiments were repeated twice.

### Rapid Diagnosis of Specific Mutations in ACCase- and ALS Genes

The mutations in ACCase or ALS enzyme were analyzed by Loop-Mediated Isothermal Amplification (LAMP) assay. The specific probes to detect single nucleotide polymorphisms (SNPs) at specific position in ACCase (GenBank accession AJ310767) or ALS (AJ437300) enzymes were designed using DNA sequences from *A. myosuroides*. Leaf samples of three putative HR *L. rigidum* (ES-resistant 1 to 3) and susceptible (ES-susceptible 1) populations (BBCH 13-15) were collected from plants grown as described previously, with five pots per population. Five leaf blades (∼0.5 cm/leaf blade) from five individual plants were pooled together for testing the mutations. For each population, three biological replicates were assayed per probe. 750 μL of alkaline-polyethylene glycol solution (pH 13.4) was added to leaf samples and the samples were shaken for 30 s to extract DNA. 5 μL of DNA samples were added to the reaction contained 15 μL Isothermal MasterMix (ISO-001, OptiGene) and 5 μL of specific probes (OptiGene)for each type of mutation in ALS (Trp 574) or ACCase (Ile-1781, Trp-2027, Ile-2041, and Asp-2078) protein. The LAMP reactions were performed with a Genie III (OptiGene) machine. The reactions were activated at 65°C for 30 min followed by an isothermal step at 95°C for 2 min and annealing from 40 to 70°C. The SNP at each position was identified by the shifting of melting temperature of the product compared to those of wild type form using synthetic DNA constructs (g-block) (Supplementary Table 1). The tests were done using pooled samples. As such, the mutation frequency within the population was not determined.

### Non-target-Site Resistance Testing: Analysis of a Biomarker Protein Using Enzyme-Linked Immunosorbent Assay (ELISA)

The level of LrGSTF1 protein was analyzed in total protein extract from leaf tissue. Leaf tissue from five individual plants (BBCH 13–15) were pooled into one biological replicate with five biological replicates used for quantification. Total protein was extracted from frozen ∼100 mg (fresh mass) of leaf tissue by grinding in liquid Nitrogen before adding 900 μL extraction buffer (100 mM Tris–HCl, 150 nM NaCl, 5 mM EDTA, 5% glycerol, 2% PVPP, and 10 mM DTT; pH 7.5). The leaf suspensions were incubated on ice for 10 min before centrifugation at 12,000 ×*g*, 4°C for 30 min. The supernatants were collected and recentrifuged for 15 min. The total protein concentration of clear supernatants was quantified by the Bradford assay following the manufacturer’s protocol (Bio-Rad Laboratories, United Kingdom). The concentration of the polypeptides recognized by the anti- *AmGSTF1*-serum from *L. rigidum* protein samples was determined using ELISA (Davies et al., 2020). The concentration of the homolog LrGSTF1 protein in *L. rigidum* samples (five biological replicates per population) were calculated using a standard curve prepared using recombinant *AmGSTF1* protein (non-linear regression four parameters logistic analysis; Graphpad PRISM v.8.2).

### P450: Herbicide Synergism of Malathion in Whole Plant Studies

To study the potential role of CYP450 in NTSR by enhanced metabolism in these HR *L. rigidum* populations, a CYP450 inhibitor was applied in post-emergence to assess its synergism with the herbicide prosulfocarb. All populations were sprayed at the BBCH stage of 13-21 (Zadoks et al., 1974) with prosulfocarb (Auros, Syngenta Agro, 80%) at 0, 400, 800, 1600, 3,200 (field rate), and 6,400 g ha^–1^. One hour before herbicide treatment, all populations were sprayed with either 0 or 1,000 g a.i. ha^–1^ of the organophosphate insecticide malathion {[(dimethoxyphosphinothioyl)-thio] butanedioic acid diethyl ester}. Previous research has shown that 1,000 g a.i. ha^–1^ is around the maximum dose of malathion that can be used without adverse effects in *L. rigidum* (Christopher et al., 1994; Preston et al., 1996; Yu et al., 2009). A total of four replicates were included at each dose, with non-treated plants used as controls and prosulfocarb applied as described above. Percentage of survival was estimated in each case, and plants were harvested (above ground) and the fresh weight measured. Experiments were repeated twice.

It is important to note that thiocarbamates such as prosulfocarb are initially CYP450-mediated oxidized to the sulfoxide, the active form of the herbicide (Fuerst, 1987). To assess the role of CYP450 in activating this herbicide, all populations were applied with a pre-emergence treatment after a pre-treatment with malathion in a second run of experiments. Populations were sprayed at the BBCH stage of 00-10 (Zadoks et al., 1974) with prosulfocarb at 0, 1,200, 2,400, and 4,000 g ha^–1^ (maximum field rate). One hour before herbicide treatments, all populations were treated with either 0 or 1,000 g a.i. ha^–1^ of malathion. Experimental design and applications were as described previously. Only survival was estimated. Population ES-resistant 3 was not included in this study due to limited seed availability.

### Statistical Analysis

Sensitivity was assessed for each *L. rigidum* population at each herbicide treatment by expressing the percentage of reduction in measured fresh biomass as compared to that determined in the untreated controls. The requirement of homogeneity of variance was checked by visual inspection of the residual plots and residuals were analyzed using the Shapiro–Wilk Test. When required, data were previously transformed. A two-way ANOVA was performed on untransformed data for each herbicide screen (ACCase inhibitors, ALS inhibitors, chlortoluron, prosulfocarb, and flufenacet plus diflufenican). Population and herbicide dose were used as factors and replicates used as an error term (block). Tukey’s honest significant difference (HSD) tests were performed to compare the percentage control of suspected resistant populations relative to the known HS plants for each species.

Differences in amounts of the AmGSTF1 were compared among populations using one-way ANOVAs followed by Tukey’s HSD test using SPSS v.24 software (IBM, Chicago, IL, United States). The assumption of homogeneity of variance for one-way was tested by Levene’s test. As the data did not fit the normal distribution, it was transformed in to log10 before doing ANOVA.

Data from dose-response experiments with a pre-treatment of malathion in POST were analyzed using a non-linear regression model. The herbicide rates required for 50% survival reduction (LD_50_), or 50% growth reduction in fresh weight of plants (GR_50_) and for 90% survival reduction (LD_90_), or 90% growth reduction (GR_90_) were calculated with the use of a four parameter logistic curve (Seefedlt et al., 1995), of the type:

$$y=c+\frac{d-c}{1+\mathrm{EXP}\,\left[b\,\left(\log\,\left(x\right)-\log\,\left(\mathrm{LD}\,50\,\mathrm{or}\,\mathrm{GR}\,50\right)\right)\right]}$$

where c = the lower limit set to 0, d = the upper limit set to 100, and b = the slope at the LD_50_ or GR_50_. In this regression equation, the herbicide rate (g a.i. ha^–1^) was the independent variable (x) and the survival (%) or plants’ fresh weight expressed as percentage of the untreated control were the dependent variables (y). The resistance index (RI) was computed as RI_50_ = LD_50_ or GR_50_(R)/LD_50_ or GR_50_(S) or as RI_90_ = LD_90_ or GR_90_(R)/LD_90_ or GR_90_(S), both for survival or fresh weight reduction. Repetitions from the dose-response experiments were pooled due to lack of statistical differences between them. Non-linear regressions were carried out with the use of Sigmaplot 11.00 (Systat Software, 2007).

Survival data from the experiment with a pre-treatment of malathion in POST was analyzed with a three-way ANOVA. Population, inhibitor and herbicide dose were used as factors and replicate used as an error term (block). Tukey’s honest significant difference (HSD) tests were performed to compare treatment means.

## Results

### Herbicide Screening

**FIGURE 1:**
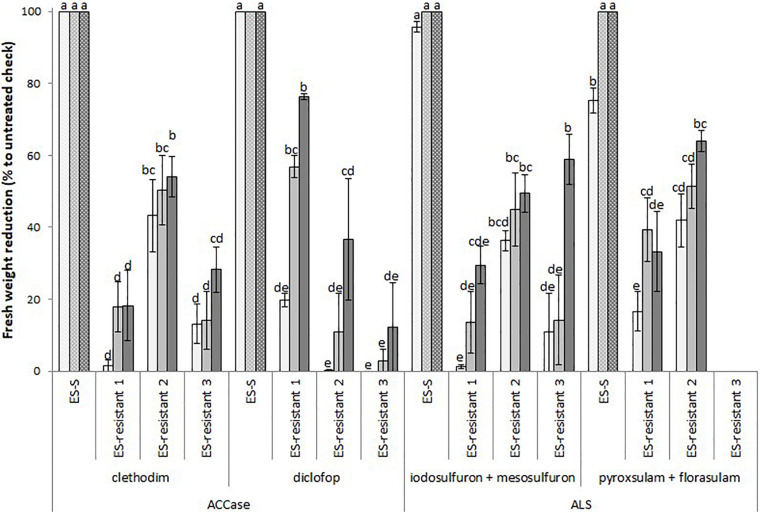
Mean reduction in foliage fresh biomass relative to untreated control for multiple HR populations of *Lolium rigidum* to ACCase (group 1, HRAC/WSSA) and ALS inhibitors (group 2). Populations ES-resistant 1 to 3 compared to a known sensitive population (ES-S). Treated with four herbicides at three rates each (columns with different gray intensity from lowest to highest rate): two ACCase inhibitors, clethodim (24, 48, and 96 g ha^− 1^) and diclofop (180, 360, and 720 g ha^− 1^), and two ALS inhibitors, mesosulfuron (3.8, 7.5, and 15 g ha^− 1^) plus iodosulfuron (0.8, 1.5, and 3 g ha^− 1^), and pyroxsulam (9.4, 18.8, and 37.5 g ha^− 1^) plus florasulam (1.9, 3.8, and 7.5 g ha^–1^). Error bars are standard error of the mean. N.D., not determined.

All three *L. rigidum* populations used in this study were poorly controlled by both ACCase- and ALS-inhibiting herbicides. Statistical comparison of these three populations against S plants showed that they had a significantly lower percentage reduction in fresh biomass at all herbicide rates tested (Figure 1). For the ACCase inhibitors clethodim and diclofop, the fresh biomass reductions were lower than 60% in most cases, except for the ES-resistant 1 population that showed ∼80% biomass reduction when treated with the highest dose of diclofop. Similarly, the reduction in fresh biomass compared to the S population did not extend beyond 60% for the ALS inhibitors iodosulfuron + mesosulfuron and pyroxsulam + florasulam treatments. An exception was the treatment with the highest dose of pyroxsulam + florasulam in the ES-resistant 2 population (64%). No data was available for the ES-resistant 3 populations for the mixture of ALS-inhibitors pyroxsulam + florasulam. Together, these results suggested that the three resistant populations of *L. rigidum* showed enhanced tolerance to multiple chemistries of ACCase- and ALS-inhibiting herbicides.

**FIGURE 2:**
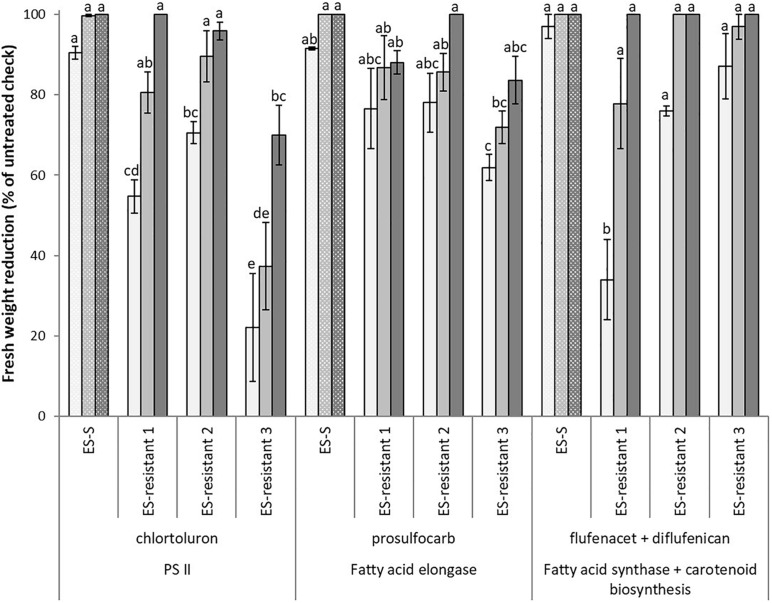
Mean reduction in foliage fresh biomass relative to untreated control for multiple HR populations of *Lolium rigidum*. Populations ES-resistant 1 to 3 compared to a known sensitive population (ES-S). Treated with three herbicides at three rates each (columns with different gray intensity from lowest to highest rate): one PS II inhibitor (group 5, HRAC/WSSA), chlortoluron (750, 1,500, and 3,000 g ha^− 1^), one fatty acid elongase inhibitor, prosulfocarb (group 15, thiocarbamates) (1,000, 2,000, and 4,000 g ha^− 1^), and one fatty acid synthase inhibitor, flufenacet (group 15, oxyacetamides) (60, 120, and 240 g ha^− 1^) plus one carotenoid synthesis inhibitor, diflufenican (group 12) (30, 60, and 120 g ha^− 1^). Error bars are standard error of the mean.

Besides enhanced resistance to ALS- and ACCase-inhibiting herbicides, poor controls in terms of fresh weight reduction (22–89%) were observed in the three HR populations when treated with the PS II inhibitor chlortoluron at 0.25 and 0.5x rates (Figure 2). While at field recommended rate (1,500 g ha^–1^), only ES-resistant 3 population showed a lower percentage of fresh weight reduction (70%) compared to the S population.

Apart from the resistance to post-emerging herbicides, the three HR populations showed a lower percentage of fresh biomass reduction at all rates of prosulfocarb treatments (62–88%), except for the highest rate tested (4,000 g ha^–1^) in the ES-resistant 2 population (Figure 2). Finally, for the formulated mixture flufenacet plus diflufenican, no significant differences were found among the populations, though reduced susceptibility was observed at the lowest rate in the ES-resistant 1 and 2 populations (34 and 76%), and also at the intermediate rate (120 + 60 g ha^–1^) in ES-resistant 1 (78%) (Figure 2).

Taken together, our results confirmed that all three HR *L. rigidum* populations studied showed very high levels of increased tolerance to ACCase- and ALS-inhibiting herbicides, as well as moderate tolerance toward PS II and Fatty acid elongase inhibitors (Table 3). Overall, the ES-resistant 3 population, showed the highest levels of increased tolerance (cross-resistance) to these four MoAs. It is noteworthy that none of the researched populations showed increased tolerance to the herbicide mixture flufenacet + diflufenican.

**TABLE 3:** Summary of the resistance profiles of one susceptible and three multiple HR populations of *Lolium rigidum* from Spain to five SoA.

	**ALS**		**ACCase**		**PS II**	**F. a. elongase**	**F. a. synthase+carotenoid**
**Population**	**Iodosulfuron+mesosulfuron**	**Pyroxsulam+florasulam**	**diclofop**	**clethodim**	**chlortoluron**	**prosulfocarb**	**Flufenacet+diflufenican**
ES-S	S	S	S	S	S	S	S
ES-R 1	R	R	R	R	MR	MR	MR
ES-R 2	R	R	R	R	MR	MR	LR
ES-R 3	R	–	R	R	MR	R	LR

*F.a., fatty acid; S, susceptible; LR, low resistant (mean efficacy below 90% only at the lowest rate); MR, moderate resistant (mean efficacies below 90% at low and medium rates); R, resistant (mean efficacies below 80% at the three rates).*

### Rapid Diagnosis of Specific Mutations in ACCase- and ALS Genes

The high level of resistance to specific chemistries of ALS or ACCase-inhibiting herbicides including mesosulfuron+iodosulfuron (SU), diclofop (APP) and clethodim (CHD) in *L. rigidum* are known to be caused by the mutations at specific position in ALS or ACCase enzymes. To assess whether these specific mutations existed in the R populations, we utilized Loop-Mediated Isothermal Amplification (LAMP) as a novel rapid diagnosis technique. Although the LAMP assay will not provide information on the specific amino acid substitution, this technology will detect a SNP in the specified position in ALS or ACCase genes within 45 min. Hence, LAMP assay can be used as a tool to monitor the emergence of SNPs.

Based on the resistance profiles, we selectively tested specific SNPs in ALS (Pro-197 and Trp-574) and ACCase (Ile-1781, Trp-2027, Ile-2041, and Asp-2078) that have been previous reported to confer resistance to SU, APP, and CHD herbicides in *L. rigidum* (Yu et al., 2007, 2008). All plants tested from ES-resistant 1 and 2 populations possessed mutations at Trp-2027 and Asp-2078 positions (but not in Ile-1781 or Ile-2041) of the ACCase gene. However, no mutations of the ACCase gene at these selected positions were found in the ES-resistant 3 population (Table 4). It was interesting that the mutation at Asp-2078 has been reported to be a specific mutation that confers resistance to clethodim (CHD) in *L. rigidum* (Yu et al., 2007). Furthermore, the mutation at Trp-2027 has been reported to confer cross-resistance between APP and CHD herbicides (Yu et al., 2007). Together, these results suggest that the mutations in the ACCase enzyme might contribute to the high resistance to APP and CHD herbicides in ES-resistant 1 and 2 populations. Whereas, tolerance to ACCase inhibiting herbicides was more likely due to NTSR in the ES-resistant 3 population.

**TABLE 4:** The mutations in ALS or ACCase genes in Spanish *Lolium rigidum* populations, one susceptible and three putative multiple herbicide resistant.

	**ALS**M		**ACCase**			
**Populations**	**Trp-574**	**Pro-197**	**Ile-1781**	**Trp-2027**	**Ile-2041**	**Asp-2078**
ES-sensitive 1	N.D.	N.R.	N.D.	N.D.	N.D.	N.D.
ES-resistant 1	N.D.	N.R.	N.D.	Mutation	N.D.	Mutation
ES-resistant 2	N.D.	N.R.	N.D.	Mutation	N.D.	Mutation
ES-resistant 3	N.D.	N.R.	N.D.	N.D.	N.D.	N.D.

*N.D., not detected; N.R., not reported. Three biological replicates were assay for each mutation.*

When *L. rigidum* populations were subjected for detecting the mutations at Pro-197 and Trp-574 in ALS genes, no plants from the three HR *L. rigidum* populations possessed mutations at Trp-574 position (Table 4), which is known to confer resistance to SU and IMI. However, we observed low and inconsistent signal which could lead to the false interpretation when the primers for detecting the SNP at Pro-197 position was used. Therefore, we excluded the results of Pro-197 from this study. It is noteworthy that the mutation at Pro-197 is known to be associated with resistance to SU such as mesosulfuron + iodosulfuron (Yu et al., 2008; Tranel et al., 2020). Although we cannot confirm the existent of Pro-197 in the R populations, it is possible, though unlikely based on reported incidence, that other mutations (Ala122, Ala205, and Asp376) could contribute to the observed resistance to SU (Murphy and Tranel, 2019; Tranel et al., 2020). Additionally, there is no report of a specific mutation in the ALS gene that confers resistance to pyroxsulam, a triazolopyrimidine (TRP), in *L. rigidum*.

### Non-target-Site Resistance Mechanism Testing

#### GSTF1: Enzyme-Linked Immunosorbent Assay (ELISA)

**FIGURE 3:**
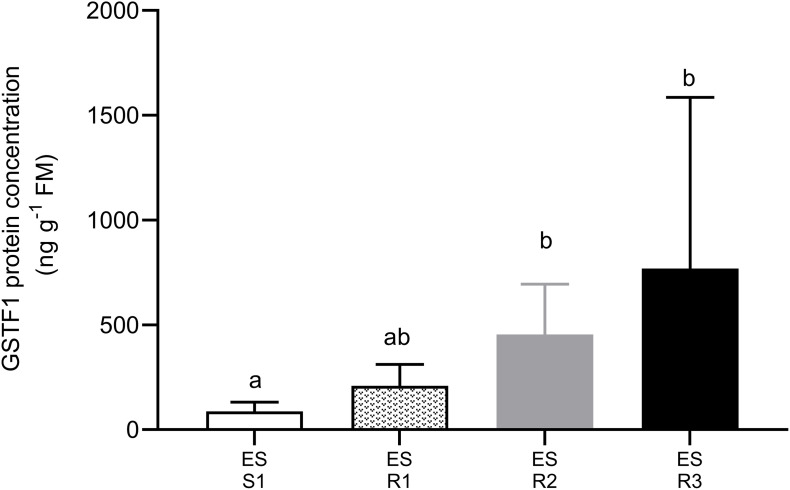
The content of the ortholog protein of *AmGSTF1* in a susceptible and three multiple HR populations of *Lolium rigidum*. Ortholog protein abundance was quantified by ELISA. Each bar represented an average (mean ± SD, *n* = 5) of protein abundance in each population. Different letters indicate significant differences among population (*P* ≤ 0.05).

To explore the possibility that NTSR could contribute to resistance to ACCase- and ALS-inhibiting herbicides and confer cross-resistance to other MoAs, the presence of the Lolium ortholog of *AmGSTF1*, a well-characterized biomarker of this type of resistance in wild grasses (Cummins et al., 2013), was assessed. Western blotting analysis showed that antisera raised to *AmGSTF1* detected a polypeptide typical of the respective GST subunit in all *L. rigidum* populations studied. The concentrations of the respective immunoreactive polypeptides were then determined by ELISA. The levels of AmGSTF1 immunoreactive polypeptides were significantly higher in the HR *L. rigidum* populations than in the S population (*F* = 5.998, *P* = 0.007). Irrespective of the presence of mutations in the ACCase or ALS genes, all HR populations showed enhanced levels of the AmGSTF1 ortholog (Figure 3). Moreover, the ES-resistant 3 population, which did not show mutation in the ACCase gene, had higher levels of the *AmGSTF1* ortholog protein together with ES-resistant 2 population, compared to the S one.

#### CYP450: Herbicide Synergism of Malathion in Whole Plant Studies

**FIGURE 4:**
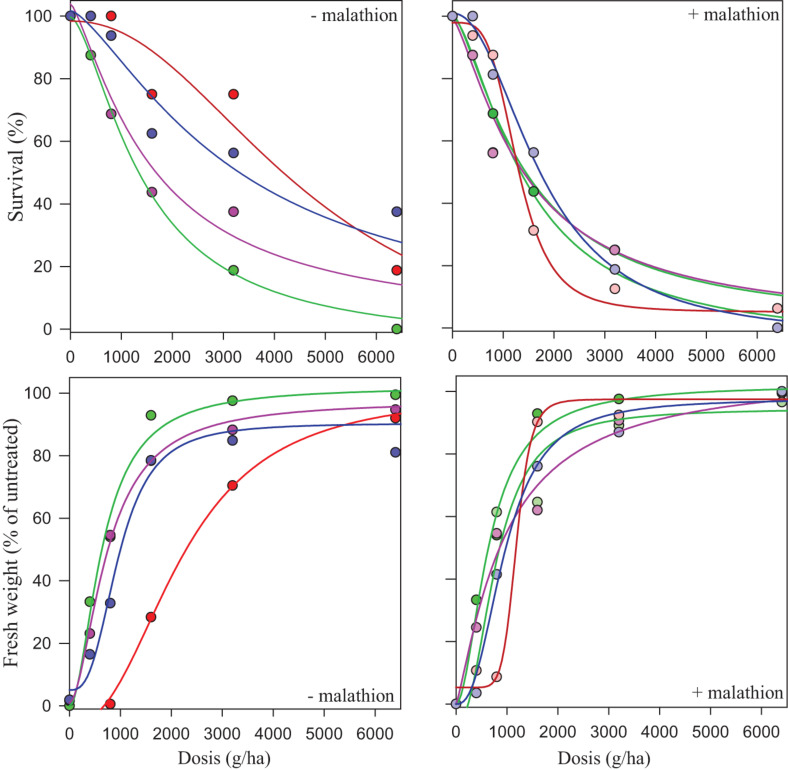
Dose-response regression curves of prosulfocarb without malathion (− malathion, left hand graphs), or with a pre-application of malathion (right hand graphs) at a dose of 1,000 g/ha (+ malathion) in susceptible (S, green lines and dots) and resistant populations ES-resistant 3 (pink lines and dots), ES-resistant 2 (blue lines and dots) and ES-resistant 1 (red lines and dots), of *Lolium rigidum*. The *y* axis shows both the percentage of survival (upper boxes) and the effect on percentage of the mean fresh weight (lower boxes) as compared with untreated control plants. Dashed (+ malathion) and solid (− malathion) lines represent predicted values derived from the regression analysis.

The CYP450 contains some of the most important detoxification enzymes that confer resistance to multiple herbicides in *L. rigidum*. To assess the involvement of CYP450, the resistance to herbicides following a pre-treatment with malathion (a CYP450s inhibitor) in the three HR populations were determined. When malathion was applied alone at 1,000 g ha^–1^, there was no effect on survival or growth in either the S or R populations. When prosulfocarb was applied post-emergence after a pre-treatment with malathion on the S population, survival and biomass were unaffected by the insecticide (Table 5 and Figure 4). RI for these two parameters, both based on LD_50_/GR_50_ or LD_90_/GR_90_, ranged between 0.99 and 1.53 for the S population (Table 5). In the presence of malathion, ES-resistant 1 and 2 populations became more susceptible to prosulfocarb (Table 5 and Figure 4); the RI_50_ for percentage of survival went down from 3.28 to 0.89 and from 2.25 to 1.22, respectively, and RI_90_, from 1.98 to 0.61 and from 3.55 to 0.97; for percentage fresh weight reduction, RI_50_ went down from 3.39 to 1.83 and from 2.88 to 0.84, respectively (Table 5). In contrast, malathion did not synergize prosulfocarb significantly in the ES-resistant 3 population. Thus, the survival, or effect on fresh weight (based on LD_50_/GR_50_ or LD_90_/GR_90_) and RI ranged between 0.98 and 2.01, with or without the CYP450 inhibitor in the ES-resistant 3 population.

**TABLE 5:** Equation parameters of the log-logistic models used to estimate dose-response regression curves (% Survival and % fresh weight of untreated control) in susceptible (S) and potential prosulfocarb resistant *Lolium rigidum* populations (ES-R 1 to 3) for prosulfocarb with (+ malathion) or without (− malathion) pre-application of malathion at 1,000 g/ha, both in post-emergence.

**Parameter**	**Biotype**	**Treatment**	**Slope^a^**	**XR_50_^b^**	**XR_90_^c^**	**RI_50_**	**RI_90_**	**R^2^**
Survival	S	− Malathion	–1.572	1440	4,381	1.00	1.00	0.999
		+ Malathion	–1.425	1426	6,704	0.99	1.53	0.980
	ES-R 3	− Malathion	–1.309	1586	8,785	1.10	2.01	0.981
		+ Malathion	–1.354	1408	7,092	0.98	1.62	0.982
	ES-R 2	− Malathion	–1.412	3237	15,558	2.25	3.55	0.973
		+ Malathion	–2.162	1764	4,254	1.22	0.97	0.998
	ES-R 1	− Malathion	–2.193	4721	8,653	3.28	1.98	0.983
		+ Malathion	–3.986	1288	2,657	0.89	0.61	0.997
Fresh weight (%)	S	− Malathion	1.883	649	1,887	1.00	1.00	0.994
		+ Malathion	2.350	777	2,852	1.20	1.51	0.968
	ES-R 3	− Malathion	1.803	943	3,740	1.45	1,98	0.999
		+ Malathion	1.241	743	2,903	1.14	1.54	0.992
	ES-R 2	− Malathion	2.902	965	5,969	1.49	3.16	0.986
		+ Malathion	2.525	961	2,545	1.48	1.35	0.995
	ES-R 1	− Malathion	2.445	2200	5,431	3.39	2.88	0.999
		+ Malathion	8.239	1188	1,594	1.83	0.84	0.997

*XR_50_ and XR_90_ are expressed as g a.i./ha of prosulfocarb. RI, resistance index as RI_50_ = XR_50_(R)/XR_50_(S) or as RI_90_ = XR_90_(R)/XR_90_(S).*

*^*a*^Slope at the XR50.*

*^*b*^XR_50_ represents LD_50_ for survival, and GR_50_ for fresh weight (%), herbicide concentration for 50% reduction of *L. rigidum* survival and fresh weight, respectively.*

*^*c*^XR_90_ represents LD_90_ for survival, and GR_90_ for fresh weight (%), herbicide concentration for 90% reduction of *L. rigidum* survival and fresh weight, respectively.*

**FIGURE 5:**
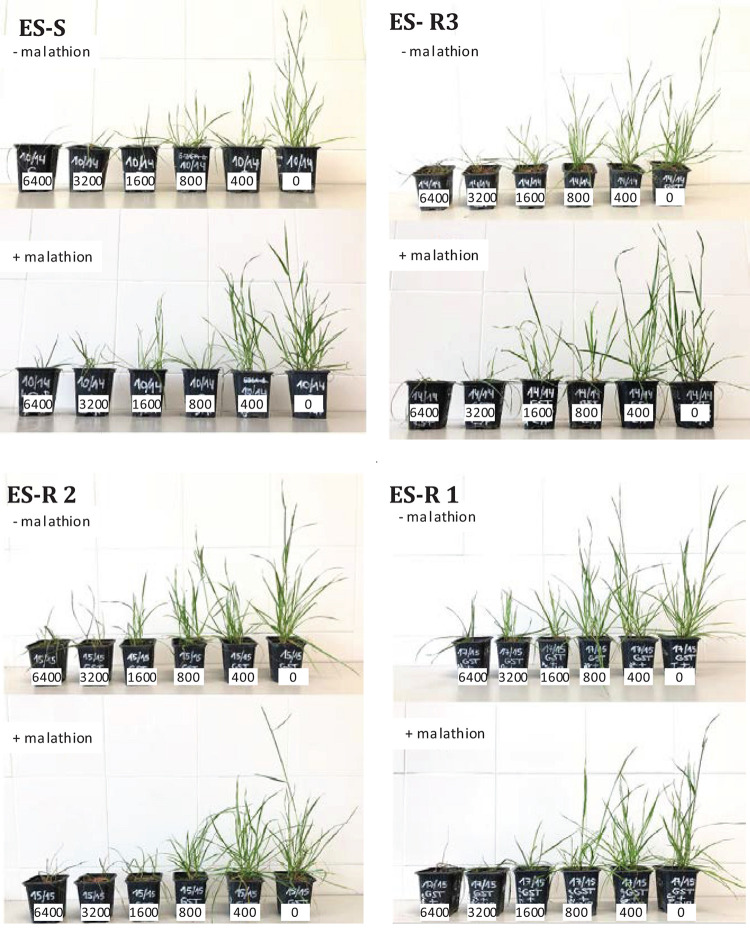
Visual injury of four *Lolium rigidum* populations 28 days after treatment with prosulfocarb (+ malathion, lower boxes), or without (− malathion, upper boxes) a pre-treatment with malathion (1,000 g a.i./ha). Prosulfocarb was applied at five rates (6,400, 3,200, 1,600, 800, 400, and 0 g a.i./ha) as arranged from the right to left hand side in each case. Left upper box, susceptible population (ES-S); left right, resistant population (ES-R 3); bottom left, resistant population (ES-R 2); bottom right, resistant population (ES-R 1).

Visual inspection of treated ES-resistant 1 and 2 plants comparing both treatments (± malathion pre-treatment), revealed that survival and growth were partially reduced in the presence of the insecticide, particularly at higher prosulfocarb rates. This partial reversion of the phenotype was not observed for the ES-resistant 3 population or the S standard population (Figure 5). This suggested that, besides the role showed by CYP450, a secondary NTSR mechanism that did not involve these enzymes was exhibited in these plants.

## Discussion

Resistance to two chemical classes of ACCase-inhibiting herbicides (group 1 HRAC/WSSA), APP (diclofop) and CHD (clethodim), and to families of ALS-inhibiting herbicides (group 2), SU (mesosulfuron + iodosulfuron) and TRP (pyroxsulam + florasulam) has been confirmed in three HR *L. rigidum* populations from Spain. Furthermore, cross-resistance, or reduced susceptibility, to other SoA including the PS II inhibitor chlortoluron (group 5) and to the fatty acid elongase inhibitor prosulfocarb (group 15) was also observed in these HR populations. These are the first confirmed reports of multiple HR and cross-resistance in *L. rigidum* to these SoA in Spain and Europe. Field-evolved resistance to different pre-emergence herbicides, including prosulfocarb, has previously been confirmed in Australia (Brunton et al., 2018). Together, these findings further emphasize the plasticity of *L. rigidum* to develop resistance mechanisms to broad spectrum of herbicides.

Non-target-site resistance is a generalist resistance mechanism that develops independently from TSR, a specialist resistance mechanism that confers resistance to only one herbicide SoA, with several grasses exhibiting both classes of resistance (Comont et al., 2020). EMR is one of the best-known mechanisms under-pinning NTSR and confers broad spectrum resistance to multiple herbicide SoA in weeds (Ghanizadeh and Harrington, 2017; Gaines et al., 2020).

Resistance to different chemical classes of ACCase-inhibiting herbicide in *L. rigidum* can result from TSR, or/and NTSR mechanisms (Takano et al., 2021). Point mutations at Trp-2027 and Asp-2078 in the ACCases of the two HR populations ESR-1 and ESR-2 were determined, with no TSR-conferring mutations found at any of the four positions tested in the HR population ES-resistant 3. Mutations at position Asp-2078 in the ACCase gene is one the most common mutation conferring TSR across a range of grass weed species. Asp-2078 is located close to the active, with mutations at this position conferring resistance to multiple chemistries acting on this enzyme (Gaines et al., 2020). Apart from Asp-2078 position, mutations in seven alternative positions in the ACCase gene are linked to TSR toward differing classes of ACCase-inhibiting herbicides in various weed species (Kaundun, 2014; Murphy and Tranel, 2019). Therefore, it is important to test for the other two ACCase positions described in *L. rigidum* (Cys2088 and Gly2096) before the final assessment of the impact of point mutations on the resistance to ACCase-inhibiting herbicides in these HR populations can be concluded. However, based on the broad resistance to herbicides acting on multiple SoA observed in the ES-resistant 3 population, it would appear most likely that NTSR was responsible for tolerance to diclofop and clethodim in these plants. Furthermore, the level of AmGSTF1 ortholog protein (LrGSTF1) was significantly elevated in this population compared to the sensitive population which further pointed to NTSR mechanisms being activated in this *L. rigidum* population (Cummins et al., 2013). Further experiments are required to identify the specific NTSR mechanisms in these plants as previous studies have shown that reduced herbicide absorption could be one of the mechanisms that confer resistance to ACCase-inhibiting herbicide in Spanish *L. rigidum* populations (De Prado et al., 2005).

The three HR populations also showed resistance toward two chemical classes of ALS-inhibiting herbicides. To test for mutations giving rise to TSR, we found no mutation at Trp-574 position in any of the three HR populations. There are four further mutations in ALS gene of *L. rigidum* that can confer resistance to herbicides acting on this enzyme, including the common mutation at Pro-197 (Yu et al., 2008; Murphy and Tranel, 2019; Tranel et al., 2020). We could not confirm whether there is the mutation at Pro-197 using LAMP assay due to the variation of the sequences in the region surrounding Pro-197 in *L. rigidum*. As LAMP assay requires six primers to detect six distinctive regions around the sequence of interest, the Pro-197 primers produced low signal when *L. rigidum* DNA was used as template. The new design of LAMP primers to detect the mutation at Pro-197 in *L. rigidum* is required for future studies. Regardless of Pro-197 mutation, the DNA sequencing of ALS gene to identify the mutations at additional positions in the ALS is required to provide conclusive evidence of the potential for TSR contributing to resistance to ALS-inhibiting herbicides in these HR populations. However, it is also probable that there are no TSR mutations in their ALS gene. As mentioned previously, considering the broad resistance to different ALS inhibiting chemistries shown by these populations, it is unlikely the presence of mutations in position Pro-197. Moreover, in north-eastern Spain, grass control was based on chlortoluron for several decades. This scenario usually drives a selection pressure linked to EMR through enhanced CYP450 expression as being the main NTSR mechanisms (Gaines et al., 2020). Subsequent selection pressure with ALS-inhibitors would not have necessarily selected for TSR, since enhanced CYP450 activity underpinning NTSR already conferred cross-resistance to multiple herbicides in these *L. rigidum* populations.

Apart from enhanced resistance to ACCase and ALS-inhibiting herbicides, the HR populations also showed moderate levels of cross-resistance, or reduced susceptibility, to the PS II inhibitor (chlortoluron) and a thiocarbamate that inhibits fatty acid elongase (prosulfocarb). As the mutation (TSR) that confer resistance to these herbicides remain largely un-recorded in grass weeds including *L. rigidum*, it is probable that NTSR/EMR would be the resistance mechanism toward these herbicides. Dose-response experiments with prosulfocarb in the presence of the CYP450 inhibitor malathion, indicated that CYP450 could potentially contribute to these reduced susceptibilities prosulfocarb observed in these HR populations. It will now be of interest to test the effect of inhibiting CYP450 via malathion using chlortoluron, a herbicide where CYP450 have already been demonstrated as being involved in EMR in *L. rigidum* populations (Gaines et al., 2020). It is noteworthy that we observed an antagonistic effect when prosulfocarb was applied pre-emergence with malathion in the whole plant experiments (Table 6). This antagonism could be due to the fact that prosulfocarb is a pro-herbicide activated by CYP450 in plants (Fuerst, 1987). Therefore, the synergism (increase sensitivity in HR populations) and antagonism effects could be accounted for by the differential inhibition of diverse CYP450 by malathion. Finally, differential expression of diverse CYP450 in roots and shoots could also contribute to this phenomenon, the inhibition of enzymes responsible of herbicide activation when soil applied or enzymes responsible of prosulfocarb degradation when foliar applied.

**TABLE 6:** Survival (%) in susceptible (S) and potential prosulfocarb resistant *Lolium rigidum* populations (ES-R 1 and 2) for prosulfocarb with (+ malathion) or without (− malathion) a pre-application of malathion at 1,000 g/ha, applied in pre-emergence.

**Dose (g a.i. ha^–1^)**			**Resistant**	
	**Malathion**	**Susceptible**	**ES-resistant 1**	**ES-resistant 2**
0	–	100 ± 0	100 ± 0	100 ± 0
	+	100 ± 0	100 ± 0	100 ± 0
1,200	–	13 ± 13	70 ± 12	50 ± 20
	+	25 ± 10	88 ± 13	70 ± 14
2,400	–	0 ± 0	50 ± 12	38 ± 13
	+	13 ± 7	75 ± 14	63 ± 24
4,000	–	0 ± 0	38 ± 13	13 ± 13
	+	0 ± 0	50 ± 20	25 ± 14

Significant factors: population, malathion, and dose; among interactions only population*dose. Therefore, there are no differences between malathion treatments within each population and dose.

Besides CYP450, previous studies have indicated that the GST enzyme superfamily can mediate EMR in *L. rigidum* toward thiocarbamate herbicides belonging to the same group (15) as prosulfocarb, through enhanced detoxification following conjugation with glutathione (GSH) (Busi et al., 2018; Dücker et al., 2019). Levels of *AmGSTF1* ortholog protein (*LrGSTF1*) were elevated in the HR populations and since this enzyme has low glutathione conjugating activity toward herbicides, its presence is linked to the increased expression of other GST (Cummins et al., 2013). As it has been reported that the tau class (U) GST (GSTU) metabolized the group 15 herbicide flufenacet in *L. rigidum* (Dücker et al., 2019), it is possible that these enzymes also contribute to EMR toward prosulfocarb and other SoA herbicides (Ghanizadeh and Harrington, 2017; Gaines et al., 2020).

The *L. rigidum* populations tested here have evolved resistance to ACCase and ALS-inhibiting herbicides and are not well-controlled by other selective herbicides. Our findings suggest that both TSR and NTSR mechanisms are present in these *L. rigidum* populations which highlights the difficulties in effectively using other selective herbicide SoA to control multiple HR populations in diverse cropping systems. Altogether, the presence of ACCase and ALS HR *L. rigidum* populations in Spain, together with the confirmed presence of glyphosate-resistant (Fernandez-Moreno et al., 2017; Fernández-Moreno et al., 2017) and flufenacet-resistant populations (Dücker et al., 2019), should alert Spanish growers to the risk of multiple herbicide resistance evolving in Spanish *L. rigidum* to most available SoA on a large scale. This study confirmed the need for resistance monitoring in *L. rigidum* in Spain and the use of non-chemical control methods and integrated weed management to be adopted as preventative strategies (Cirujeda and Taberner, 2010). The precise identification of the types of resistance and the underlying molecular mechanisms that are evolving in field populations is essential to slowing, or preventing the evolution of TSR and NTSR in *L. rigidum* as well as other weed species. As such, the detection of the ortholog of *AmGSTF1* and the LAMP assay for SNP detection are promising technologies for the rapid monitoring of TSR and NTSR in *L. rigidum*.

## Data Availability Statement

The raw data supporting the conclusions of this article will be made available by the authors, without undue reservation.

## Author Contributions

JT, JM, and AT provided the plant material and wrote the first draft. JT, JM, RE, and NO carried out the experiments. All authors contributed to the experimental design, participated in data curation and analysis, and involved in the final preparation and review of the manuscript.

## Conflict of Interest

The authors declare that the research was conducted in the absence of any commercial or financial relationships that could be construed as a potential conflict of interest.

## Supplementary Material

Supplementary Table 1 The melting temperature of a specific product from wild type or SNP of ALS or ACCase enzyme detected by LAMP assay in this study.
