# Supplementary material for: Target-Site and Non-target-Site Resistance Mechanisms Confer Multiple and Cross- Resistance to ALS and ACCase Inhibiting Herbicides in Lolium rigidum From Spain
Source: Front Plant Sci. 2021 Feb 4;12:625138. doi: 10.3389/fpls.2021.625138 (PMC7889805; doi:10.3389/fpls.2021.625138)
Supplement: Supplementary Table 1 — The melting temperature of a specific product from wild type or SNP of ALS or ACCase enzyme detected by LAMP assay in this study. [file Table_1.DOCX]

**Supplementary table** The melting temperature of a specific product from wild type or SNP of ALS or ACCase enzyme detected by LAMP assay in this study

| Probe | WT peak | SNP peak |
| --- | --- | --- |
| ALS 574 | 60°C | 52.4°C |
| ACCase 2027 | 61.7°C | 50.1°C |
| ACCase 2041 | 53.7°C | 44.4°C |
| ACCase 2078 | 62.3°C | 58.7°C |
| ACCase 2096 | 59.2°C | 48.2°C |
